# LC-MS/MS-based enzyme assay for lysosomal acid lipase using dried blood spots

**DOI:** 10.1016/j.ymgmr.2022.100913

**Published:** 2022-08-26

**Authors:** Mari Ohira, Marianne Barr, Torayuki Okuyama, Ryuichi Mashima

**Affiliations:** a Department of Clinical Laboratory Medicine, National Center for Child Health and Development, 2-10-1 Okura, Setagaya-ku, Tokyo 157-8535, Japan; b Biochemistry Department, Queen Elizabeth University Hospital, 1345 Govan Road, Govan, Glasgow G51 4TF, UK

**Keywords:** Wolman disease, Cholesteryl ester storage disorder, Lysosomal storage disorder, Enzyme assay, LC-MS/MS

## Abstract

Lysosomal acid lipase deficiency (LAL-D) (OMIM: 278000) is a lysosomal storage disorder with two distinct disease phenotypes such as Wolman disease and cholesteryl ester storage disorder (CESD), characterized by an accumulation of endocytosed cholesterol in the body. Due to the presence of multiple lipases in DBS, previous studies measured LAL enzyme activity in the presence of Lalistat-2, an established LAL-specific inhibitor (Hamilton J *et al* Chim Clin Acta (2012) 413:1207–1210). Alternatively, a novel substrate specific for LAL has been reported very recently (Masi S. *et al* Clin Chem (2018) 64:690–696). In this study, we examined the LAL enzyme activity of a Japanese population with the LAL-specific substrate using liquid chromatography-tandem mass spectrometry (LC-MS/MS)-based enzyme assay whether an affected individual can be identified among this population. To achieve this, we first performed assay validation using LC-MS/MS. Under our experimental setting, typically we obtained LAL enzyme activity for QC High (100% enzyme activity) as 261.9 ± 3.2 μmol/h/L (*n* = 5) and for QC Low as (5% enzyme activity) as 14.7 ± 0.5 μmol/h/L (*n* = 5). The percentage of coefficient of variation for interday assay for QC High was 9.6% (*n* = 4) and for QC Low was 7.9% (*n* = 4), respectively. Based on these results, we further examined the LAL enzyme activity of control Japanese population and that of affected individuals with Wolman disease and CESD. The averaged enzyme activity for control newborns, Wolman, and CESD was 123.9 ± 53.9 μmol/h/L (*n* = 131), 6.6 ± 0.9 μmol/h/L (*n* = 3), and 4.8 ± 0.3 μmol/h/L (*n* = 3), respectively. These results suggest that an LAL-D-affected individual can be readily identified by enzyme activity using LC-MS/MS-based technique.

## 1 Introduction

Lysosomal acid lipase deficiency (LAL-D) (OMIM: 278000) is a lysosomal storage disorder characterized by an accumulation of endocytosed cholesterol in the body due to the deficiency of lysosomal acid lipase (EC 3.1.1.13) [[1], [2], [3]]. The major manifestations of LAL-D involves hepatosplenomegaly, hypercholesterolemia, and elevating liver enzymes leading to an underappreciated cause of fibrosis, cirrhosis and severe dyslipidemia [4,5]. There are two disease subtypes for LAL-D. An early-onset form is called Wolman disease which failed to thrive in infants with very low enzyme activity. In contrast, a later-onset form is called as cholesterol ester storage disease (CESD) that is associated with an accumulation of cholesterol esters and triglycerides, leading to, at least in part, coronary heart disease in adults. The genotype/phenotype correlation has not been fully elucidated due to the limited number of affected individuals, but in Wolman disease, small deletion/insertion, non-sense, and splicing mutations are often found. In contrast, a half of mutations in CESD are missense. In addition, there is a well-known alternative splicing mutation involving exon 8 (E8SJM^-1G>A^) [6]. In this example, pathogenic splicing of exon 8 occurs in most cases, while normal splicing occurs about 2–4%, showing low enzyme activity in patients. The major treatment options involve cholesterol reduction therapy, liver transplantation, and enzyme replacement therapy [6]. Recently, enzyme replacement therapy has been developed, thus an identification of affected individual is the first step toward treatment [4,5].

LC-MS/MS-based enzyme assay for lysosomal storage disorders (LSDs) has been widely used for newborn screening [7,8]. This technology allows us to screen multiple disorders simultaneously, therefore, Pompe, Fabry, and mucopolysaccharidosis type I (MPS I) are usually screened [[9], [10], [11]]. The assay mixture includes a substrate for the enzyme, an internal standard, and a buffer with acidic pH. For LAL enzyme assay, an inclusion of Lalistat-2, a specific inhibitor for LAL, is essential when 4MU-palmitate is used as the substrate, otherwise selective measurement of LAL enzyme activity in DBS is not achieved [[12], [13], [14]]. More recently, a specific substrate for LAL enzyme has discovered [12]. This substrate enables direct measurement of LAL enzyme activity in DBS and opens a possibility for its application to screening. In this study, we reported the LAL enzyme activity in a dried blood spot (DBS) of a Japanese population and that of disease-confirmed cases using LC-MS/MS-based technique.

## 2 Experimental procedure

### 2.1 Materials

Lysosomal Acid Lipase Activity MaxSpec(R) Assay Kit (Item No. 24854) was purchased from Cayman Chemical (Ann Arbor, MI). Acetonitrile was purchased from Fischer Scientific (Tokyo, Japan). HPLC-grade ethyl acetate (#057–03371) was obtained from FujiFilm Wako Pure Chemical Corporation (Tokyo, Japan). Deionized water was obtained from a Milli-Q water system from Millipore (Milford, MA). Formic acid was purchased from Kanto Chemical (Tokyo, Japan). The other reagents used in this study were of the highest grade commercially available.

### 2.2 Ethical approval

This study was approved by the Institutional Research Board of the National Center for Child Health and Development, Tokyo, Japan.

### 2.3 DBS

Deidentified DBS from neonate born between in June 2018 to August 2018 at the National Center for Child Health and Development, Tokyo, Japan, was used. Positive controls including three Wolman disease patients and three CESD patients were provided by Dr. John Hamilton (Biochemistry Department, Queen Elizabeth University Hospital, Glasgow, UK). Diagnosis has been performed by separate method and patient record was not disclosed at the time of this study.

### 2.4 Enzyme reaction for LAL assay

To extract enzyme in aqueous solution, a 3-mm punch of DBS was incubated with water (200 μL) in a 96-well plate (#AB-0796, ThermoFischer, Tokyo, Japan) at 200 rpm for 1 h at 25 °C. Following incubation, an aliquot (10 μL) was transferred to a single well of a 96-well deep well plate (#260252, ThermoFischer). Then, an assay mixture (30 μL) containing a substrate (0.3 mM) and an internal standard (5.03 μM) was added and the plate was centrifuged at 700 ×*g* for 5 min (PlateSpinII, Kubota, Tokyo, Japan) to ensure all liquid is at the well bottom. Subsequently, the plate was sealed with an aluminum seal (#9792, 3 M, Tokyo, Japan) and incubated using an orbital shaker model MB100-4A (Aosheng, China) at 400 rpm for 3 h at 37 °C.

### 2.5 Sample preparation for LC-MS/MS measurement

After 3 h of incubation, the reaction was terminated by adding 80 μL of deionized water followed by ethyl acetate (400 μL). Then, the sample was mixed by pipetting 10 times and centrifuged at 700 ×*g* for 5 min at room temperature. Next, the upper organic layer (300 μL) was transferred to a 96-well plate and then organic solvent was evaporated under a gentle nitrogen stream generated by a nitrogen generator model ANW3-009TMM-PCO (Nihonseiki Co.,Ltd., Osaka, Japan) using a 96-well plate temperature controller (model NDG962, Nissin Rika, Tokyo, Japan). Subsequently, a mixture of acetonitrile/water/formic acid (80,20:0.1, 200 μL) was added and mixed several times. Finally, the plate was sealed with a plate sealing plastics RAPID SlitSeal (BioChromato, Kanagawa, Japan) and placed in an autosampler at 10 °C for LC-MS/MS analysis.

### 2.6 Quantification of enzyme product using LC-MS/MS

An extracted enzyme product and an internal standard were separated on an ACQUITY CSH C18 column (2.1 × 30 mm, 1.7 μm) over 2 min at a flow rate of 0.6 mL/min with gradient elution. Chromatography was performed using binary mobile phases: mobile phase A contained 0.2% formic acid in water/acetonitrile (95/5) and mobile phase B contained 0.2% formic acid in acetonitrile. For the separation of enzyme product and other components, the percentage of mobile phase B was programmed as: 20% for 0.0–0.1 min; 20–100% for 0.1–1.0 min; 100% for 1.0–1.5 min; 20% for 1.51–2.0 min. An aliquot (5 μL) of the reconstituted sample was injected onto LC-MS/MS using a partial needle-fill method. The enzyme product and internal standard were detected on a Xevo TQ-S micro mass spectrometer (Waters, Milford, MA) using ESI-positive mode equipped with an ACQUITY H-class UPLC (Waters). The data were analyzed using data analysis software MassLynx (V4.1, Waters). Detailed analytical conditions including instrumental settings and a list of MRM table were described in Supplementary Tables 1–2.

### 2.7 Statistics

The data are expressed as mean ± standard deviation as indicated. The statistical significance of differences of the mean values from the two groups was determined by Student's *t*-test. A difference of *P* < 0.05 was considered as statistically significant.

## 3 Results

### 3.1 Assay validation

**Fig. 1:**
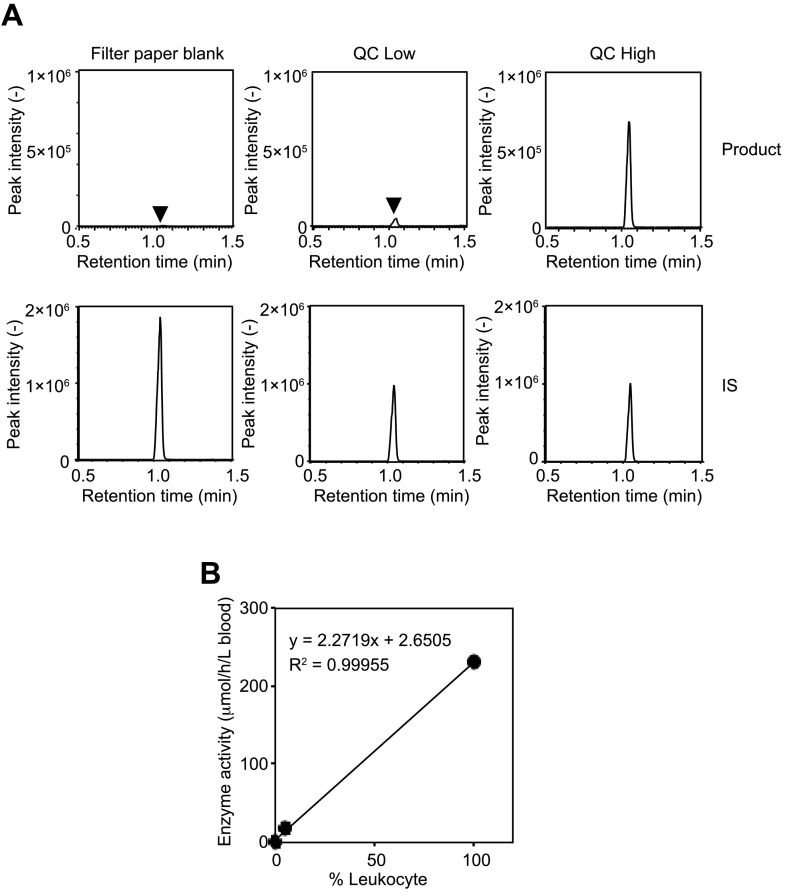
Assay validation of LAL enzyme assay with LC-MS/MS using QC specimens. (A) Chromatograms for enzyme product (Upper) of filter paper blank (Left), QC Low (Middle), and QC High (Right) were shown. Chromatograms for IS (Lower) of filter paper blank (Left), QC Low (Middle), and QC High (Right) were also shown. QC High and QC Low contained 100% and 5% of leukocytes. (B) A representative calibration curve for LAL enzyme activity.

In order to perform assay validation, first we optimized several parameters such as collision voltage and cone voltage for further assay (Supplementary Fig. 1). Then, we examined chromatographic conditions that can be applicable to high-through put assay. Eventually, we were able to find an appropriate condition that separates a peak of LAL enzyme reaction product from other impurities. Under this experimental setting, we were able to detect a peak for the enzyme reaction product and for the internal standard, both of which migrated at the same retention time (Fig. 1A). The observed LAL enzyme activity was proportional to the amount of enzyme (Fig. 1B). Next, we examined the intraday and interday assay validation using this established method. As shown in Table 1, the typical LAL enzyme activity for QC High (100% enzyme activity) was 261.9 ± 3.2 μmol/h/L (*n* = 5) and QC Low (5% enzyme activity) was 14.7 ± 0.5 μmol/h/L (*n* = 5), respectively. In this case, the CV (%) values for QC High was 1.2% and QC Low was 3.5%, respectively (Table 1). Then, we further extend this examination to perform interday assay validation. We found that CV (%) values of enzyme activity in QC High was 9.6% (*n* = 4) and in QC Low was 7.9% (*n* = 4), respectively (Table 2).

### 3.2 LAL enzyme activity in DBS

**Fig. 2:**
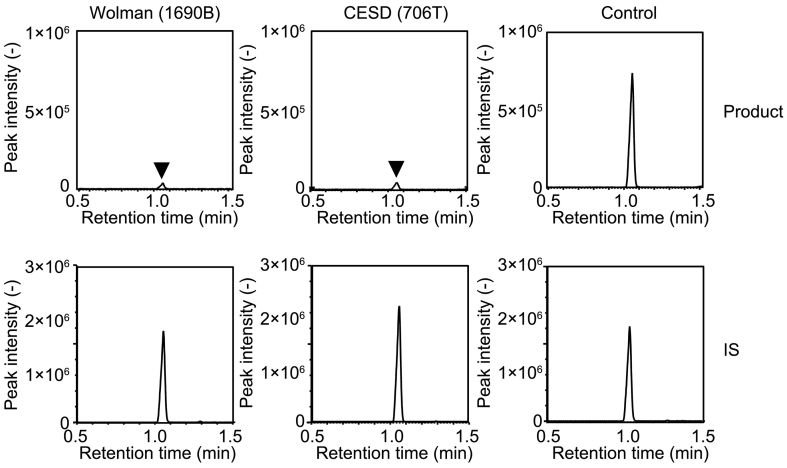
Chromatograms for clinical samples. Chromatograms of LAL enzyme reaction product (Upper) and IS (Lower) obtained from an individual with Wolman disease (Left), CESD (Middle), and of control (Right) were shown.

**Fig. 3:**
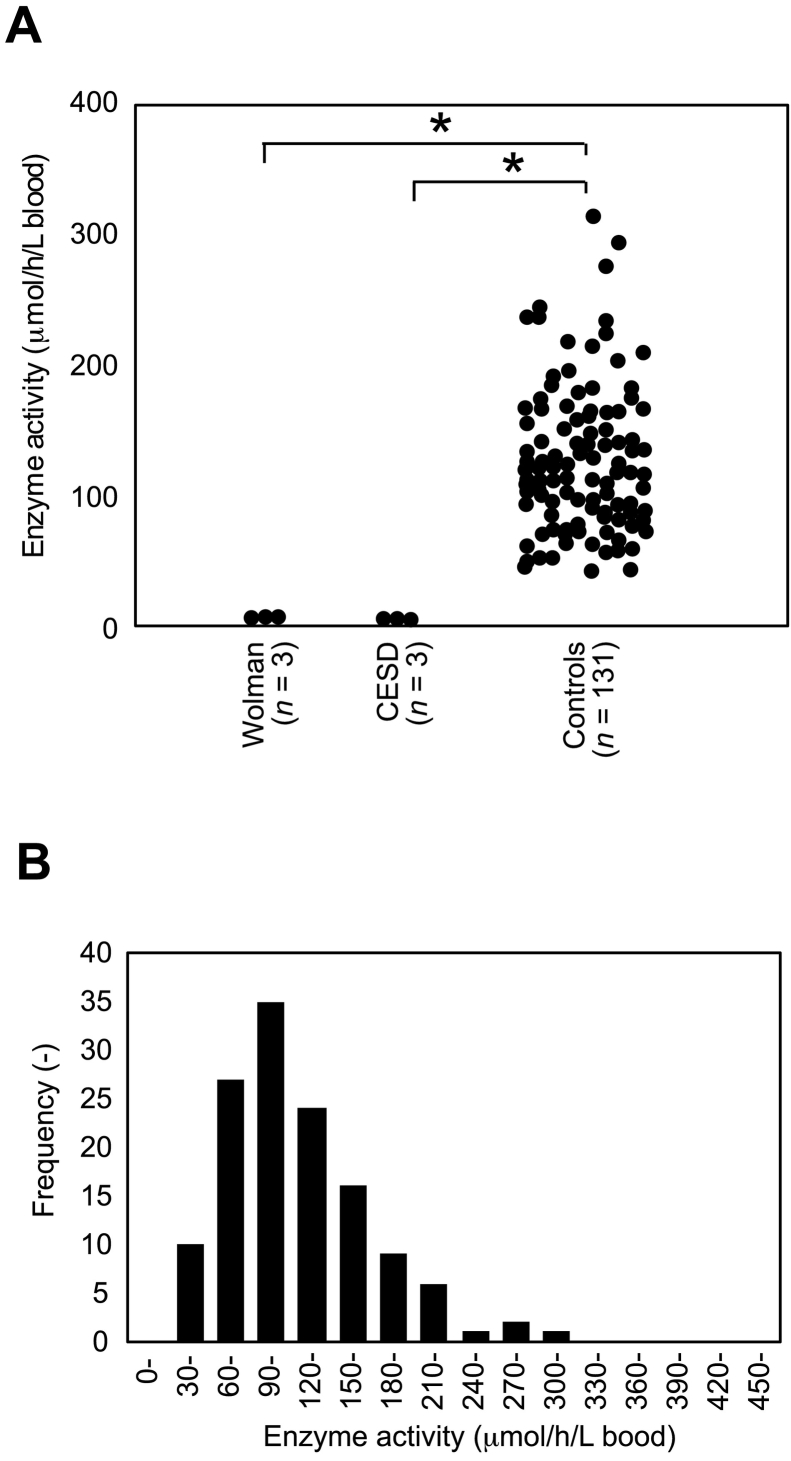
Summary of LAL enzyme activity in a Japanese population. (A) Enzyme activity of newborns and disease-confirmed specimens. The number of specimens (*n*) for Wolman disease, CESD, and controls was 3, 3, and 131, respectively. (B) Histogram of LAL enzyme activity in this study. Note that a population between 90 and 120 μmol/h/L blood of LAL enzyme activity showed most frequent (*n* = 35). The mean ± SD, median, minimum, and maximum activity was 123.9 ± 53.9 μmol/h/L, 112.7 μmol/h/L, 42.0 μmol/h/L, and 315.2 μmol/h/L, respectively.

Then, we examined the levels of LAL enzyme activity of control Japanese newborns, as well as that of confirmed cases with Wolman disease and CESD. Under this assay condition, the peak of enzyme reaction product of LAL enzyme was readily detectable in control specimen, while that in Wolman- and CESD-affected individuals was marginal (Fig. 2). The summary of enzyme activity was shown in Fig. 3. Overall, the enzyme activity of LAL in control, Wolman disease, and CESD were 123.9 ± 53.9 μmol/h/L (*n* = 131), 6.6 ± 0.9 μmol/h/L (*n* = 3), and 4.8 ± 0.3 μmol/h/L (*n* = 3) (Mean ± SD), respectively (Fig. 3A). The median, minimum, and maximum value of enzyme activity (μmol/h/L) in control were 112.7, 42.0, and 315.2, whereas that in Woman and CESD (μmol/h/L) were 7.1, 5.5, 7.1 and 4.7, 4.5, 5.1, respectively. We found 35 specimens in the range of enzyme activity between 90 and 120 μmol/h/L as the most frequent population, demonstrating that this histogram showed a single peak of distribution across all measured enzyme activity (Fig. 3B).

**Table 1:** Intraday assay validation.

Sample	Enzyme activity	CV	*n*
	(μmol/h/L blood)	(%)	
	Mean ± SD		
Filter paper blank	1.0 ± 0.1	5.6	5
Low	14.7 ± 0.5	3.5	5
High	261.9 ± 3.2	1.2	5

**Table 2:** Interday assay validation.

Sample	Enzyme activity	CV	*n*
	(μmol/h/L blood)	(%)	
	Mean ± SD		
Filter paper blank	1.2 ± 0.7	62.1	4
Low	15.7 ± 1.2	7.9	4
High	252.9 ± 24.2	9.6	4

## 4 Discussion

In 2012, a milestone study for LAL-D assay was performed by Hamilton J. *et al* that LAL enzyme activity was first measured in the presence of Lalistat-2, a specific inhibitor for LAL enzyme [13]. This method has been used by several studies and its efficacy for diagnostic has been demonstrated [[13], [14], [15]]. More recently, a new substrate that specific for LAL enzyme has been discovered [12]. The accumulation of enzyme product may be quantified using both LC-MS/MS and fluorometer. An LC-MS/MS-based method is an emerging technique which has been used in newborn screening [7,[9], [10], [11]]. In this study, we examined the efficacy of LC-MS/MS-based LAL enzyme assay by performing a validation study and by quantifying a small batch of DBS of Japanese neonate.

Compared to other published data for LAL enzyme activity, our mean enzyme activity in controls appeared to be almost a half value of them [12,13]. At this stage, the exact reason for this remains unclear. In fact, similar variation of enzyme activity in clinical specimen might be occasionally observed. For example, enzyme activity for Pompe disease, a lysosomal storage disorder with a deficiency of α-glucosidase, in newborn screening in several studies show a similar variation in different location [9,11]. Under our assay condition, the levels of LAL enzyme activity in Wolman- and CESD-affected individuals appeared to be comparable to previous data (Table 3) [12,13]. Thus, we considered that this assay readily distinguishes the populations between controls and affected individuals.

**Table 3:** Comparison of LAL enzyme activity in DBS.

Investigator	Substrate	Method	Enzyme activity (μmol/h/L)			References
			minimum – maximum (n)			
			Control	Wolman	CESD	
Hamilton J et al.	4MU-palmitate	Fluorometry	161.3–741.9 (*n* = 140)^a^	Not reported	<9.7 (*n* = 11)^a^	[13]
Masi S et al.	P-PHMC	LC-MS/MS	211.7–665.1 (*n* = 15)^b^	0.8–22.2 (*n* = 6)^c^		[12]
Ohira M et al.	P-PHMC	LC-MS/MS	42.0–315.2 (*n* = 131)	5.5–7.1 (*n* = 3)	4.5–5.1 (*n* = 3)	This study

a , Recalculated by authors: originally reported as 0.50–2.30 and < 0.03 nmol/punch/h for control and CESD [13]. ^b^, Children <5 years [12]. ^c^, LAL-D patients [12].

We also noticed that the LAL enzyme activity of Wolman individuals is similar to that of CESD ones in our data. Generally, it is widely believed that the enzyme activity of LAL in Wolman is lower than that of CESD [6]. This idea is further supported by an *in vitro* study showing that an enzyme with little or no activity has mutation that found in Wolman disease whereas that with 1–5% activity has mutation found in CESD [16]. However, it should be noted that most LAL assay methods use different substrate and reaction conditions. The situation has been improved since Lalistat-2 has been used [13]. The use of LAL-specific substrate may further provide more accurate measurement of enzyme activity [12]. Thus, it would be interesting to examine whether the population of Wolman and CESD may be distinguished based on enzyme activity by LC-MS/MS in further study.

In conclusion, we performed an assay validation for LAL enzyme assay using LC-MS/MS. The CV (%) values for intraday and interday assay was within 20%, demonstrating that this assay is applicable to clinical diagnostics. We further examined LAL enzyme activity with a small Japanese population and have found that no apparent low enzyme activity was identified in this population. Because this assay directly quantifies LAL enzyme activity with high robustness, thus an implementation of newborn screening based on this technique will be expected.

The following are the supplementary data related to this article.

Supplementary Fig. 1 Changes in peak intensity of internal standard Supplementary Fig. 1

## Declaration of Competing Interest

The authors declare that there is no conflict of interest.

## Data Availability

No data was used for the research described in the article.

## References

[1] Jones S.A., Valayannopoulos V., Schneider E., Eckert S., Banikazemi M., Bialer M., Cederbaum S., Chan A., Dhawan A., Di Rocco M., Domm J., Enns G.M., Finegold D., Jay Gargus J., Guardamagna O., Hendriksz C., Mahmoud I.G., Raiman J., Selim L.A., Whitley C.B., Zaki O., Quinn A.G. (2016). Rapid progression and mortality of lysosomal acid lipase deficiency presenting in infants. Genet. Med..

[2] Željko R., Guardamagna O., Nair D., Soran H., Hovingh K., Bertolini S., Jones S., Ćorić M., Calandra S., Hamilton J., Eagleton T., Ros E. (2014). Lysosomal acid lipase deficiency--an under-recognized cause of dyslipidaemia and liver dysfunction. Atherosclerosis..

[3] James J.M. (2017). Managing cardiovascular risk in lysosomal acid lipase deficiency. Am. J. Cardiovasc. Drugs.

[4] Simon A.J., Caro R.S., Quinn A.G., Friedman M., Marulkar S., Ezgu F., Zaki O., Gargus J.J., Hughes J., Plantaz D., Vara R., Eckert S., Arnoux J.-B., Brassier A., Quan Sang K.-H.L., Valayannopoulos V. (2017). Survival in infants treated with sebelipase Alfa for lysosomal acid lipase deficiency: an open-label, multicenter, dose-escalation study. Orphanet J. Rare Dis..

[5] Burton B.K., Balwani M., Feillet F., Baric I., Burrow T.A., Grande C.C., Coker M., Consuelo-Sanchez A., Deegan P., Di Rocco M., Enns G.M., Erbe R., Ezgu F., Ficicioglu C., Furuya K.N., Kane J., Laukaitis C., Mengel E., Neilan E.G., Nightingale S., Peters H., Scarpa M., Schwab K.O., Smolka V., Valayannopoulos V., Wood M., Goodman Z., Yang Y., Eckert S., Rojas-Caro S., Quinn A.G. (2015). A phase 3 trial of sebelipase alfa in lysosomal acid lipase deficiency. N. Engl. J. Med..

[6] Bernstein D.L., Hülkova H., Bialer M.G., Desnick R.J. (2013). Cholesteryl ester storage disease: review of the findings in 135 reported patients with an underdiagnosed disease. J. Hepatol..

[7] Gelb M.H., Scott C.R., Turecek F. (2015). Newborn screening for lysosomal storage diseases. Clin. Chem..

[8] Gelb M.H., Lukacs Z., Ranieri E., Schielen P.C.J.I. (2019). Newborn screening for lysosomal storage disorders: Methodologies for measurement of enzymatic activities in dried blood spots. Int. J. Neonatal. Screen..

[9] Scott C.R., Elliott S., Buroker N., Thomas L.I., Keutzer J., Glass M., Gelb M.H., Turecek F. (2013). Identification of infants at risk for developing Fabry, Pompe, or mucopolysaccharidosis-I from newborn blood spots by tandem mass spectrometry. J. Pediatr..

[10] Wasserstein M.P., Caggana M., Bailey S.M., Desnick R.J., Edelmann L., Estrella L., Holzman I., Kelly N.R., Kornreich R., Kupchik S.G., Martin M., Nafday S.M., Wasserman R., Yang A., Yu C., Orsini J.J. (2019). The New York pilot newborn screening program for lysosomal storage diseases: report of the first 65,000 infants. Genet. Med..

[11] Liao H.-C., Chiang C.-C., Niu D.-M., Wang C.-H., Kao S.-M., Tsai F.-J., Huang Y.-H., Liu H.-C., Huang C.-K., Gao H.-J., Yang C.-F., Chan M.-J., Lin W.-D., Chen Y.-J. (2014). Detecting multiple lysosomal storage diseases by tandem mass spectrometry--a national newborn screening program in Taiwan. Clin. Chim. Acta.

[12] Masi S., Chennamaneni N., Turecek F., Ronald Scott C., Gelb M.H. (2018). Specific substrate for the assay of lysosomal acid lipase. Clin. Chem..

[13] Hamilton J., Jones I., Srivastava R., Galloway P. (2012). A new method for the measurement of lysosomal acid lipase in dried blood spots using the inhibitor Lalistat 2. Clin. Chim. Acta.

[14] Lukacs Z., Barr M., Hamilton J. (2017). Best practice in the measurement and interpretation of lysosomal acid lipase in dried blood spots using the inhibitor Lalistat 2. Clin. Chim. Acta.

[15] Reynolds T.M., Mewies C., Hamilton J., Wierzbicki A.S. (2018). Identification of rare diseases by screening a population selected on the basis of routine pathology results-the PATHFINDER project: lysosomal acid lipase/cholesteryl ester storage disease substudy. J. Clin. Pathol..

[16] Saito S., Ohno K., Suzuki T., Sakuraba H. (2012). Structural bases of Wolman disease and cholesteryl ester storage disease. Mol. Genet. Metab..

