# Supplementary figures and images for: LC-MS/MS-based enzyme assay for lysosomal acid lipase using dried blood spots
Source: Mol Genet Metab Rep. 2022 Aug 26;33:100913. doi: 10.1016/j.ymgmr.2022.100913 (PMC9440593; doi:10.1016/j.ymgmr.2022.100913)

**A**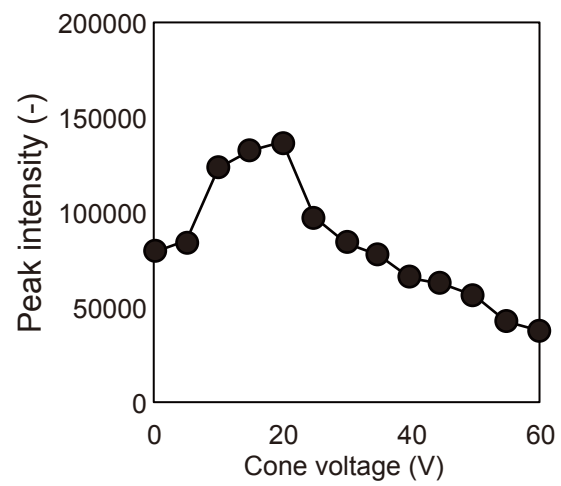**B**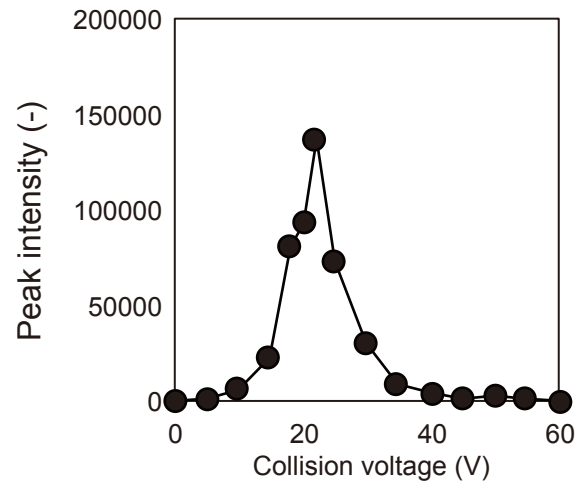

Supplement: Supplementary Fig. 1 — Changes in peak intensity of internal standard [file mmc1.pdf]
